# Comparison and Correlation Analysis of Immune Function and Gut Microbiota of Broiler Chickens Raised in Double-Layer Cages and Litter Floor Pens

**DOI:** 10.1128/spectrum.00045-22

**Published:** 2022-06-29

**Authors:** Bochen Song, Shaojia Yan, Peng Li, Guang Li, Mingkun Gao, Lei Yan, Zengpeng Lv, Yuming Guo

**Affiliations:** a State Key Laboratory of Animal Nutrition, College of Animal Science and Technology, China Agricultural University, Beijing, China; b Department of Animal Science, Shandong Agricultural University, Taian, China; c Shandong New Hope Liuhe Group Co., Ltd., Qingdao, China; University of Georgia

**Keywords:** gut microbiota, immune function, rearing system, broiler chicken

## Abstract

This study aimed to compare the immune function and gut microbiota between double-layer caged and litter floor pen-raised broiler chickens. Eighty meaty male chicks were selected and divided into cage group and litter floor group, with 20 replicates in each group. The broilers were raised in the same chicken house. The rearing density of the two rearing systems was same. The broilers were sampled on days 13 and 34. The results showed that compared with the cage group, the litter floor broilers had worse growth performance (23.24% increase in feed conversion ratio) in the early stage; better slaughter performance at day 42; stronger peripheral immune function (including higher lysozyme activity, T-cell ratio, Th-cell ratio, Tc-cell ratio, CD4/CD8, IL-10, B-cell ratio, IgG and IgA levels; and spleen immune-related gene expression); and stronger intestinal immune function (including higher ileum *CD80*, *AvBD2*, *Mucin2*, *NF-κB*, *IL-8*, *IFN-γ/IL-4*, and *IgA* mRNA expression levels and ileal mucosa sIgA levels). Compared with the cage group, the alpha diversity of ileum microbiota of the litter floor broilers was higher, and the relative abundance levels of litter breeding bacteria (*Facklamia*, *Globicatella*, and *Jeotgalicoccus*) and potential pathogenic bacteria (Streptococcus and Staphylococcus) were higher (*P < *0.05). Through Spearman correlation analysis, it was found that enriched microbes in the ileum of litter floor broilers were positively correlated with immune function. In summary, compared with cage broilers, litter floor broilers had more potential pathogenic bacteria and litter breeding bacteria in the ileum, which may be one of the important reasons for the stronger immune function status.

**IMPORTANCE** In China, the three-dimensional rearing system (cage) for broilers has gradually become a trend. In production, it was found that the incidence of disease in broiler chickens raised in cage systems was significantly higher than that of ground litter. Given that broilers raised on ground litter systems may be exposed to more environmental microbes, it is important to understand whether the rearing environment affects the function and status of the host immune system by altering the gut microbiota. In this study, rearing environment-derived gut microbes associated with stronger immune function in ground litter broilers were provided, which will provide new insights into strategies to target gut microbes to promote immune function and status in broilers raised in cages.

## INTRODUCTION

The immune system is the “army” that protects animal life, playing defense, surveillance, and self-stabilization functions. The strength of immune function directly determines the health of the animal body. The development of intestinal microbiota and the immune system are inseparable. Early microbial colonization plays an important role in many intestinal functions, including digestion, metabolic reactions, and nutritional effects. Intestinal microbiota also affects the development and maturation of the host’s innate and acquired immune systems (1, 2).

In recent years, to meet the increasing demand for chicken meat and improve the efficiency of poultry meat output, the chicken industry has gradually moved toward intensive and large-scale development in China. Cage systems with a large feeding capacity and high space utilization have gradually replaced traditional ground litter systems in China. Broilers raised under the ground litter system excrete feces on the litter, and the microbes in the feces can grow in large numbers in the warm litter, with the result that ground litter broilers can come into contact with many environmental microbes during the growth process. The cage system separates broiler chickens from manure, reducing the chance of chickens coming into contact with environmental microbes. However, proper microbial colonization is important for a well-functioning immune system. For example, the gut microbiota may be involved in the direction of class switching in B cells (3), and it has been shown that the gut microbiota can affect the production of mucosal antibodies (4). In addition, microbial colonization is important for the induction of regulatory T cells and T-cell homeostasis in the intestine (5–7). The interaction between the microbiota and the immune system explains to a certain extent the importance of exposure to microbiota for immune development and immune response. It has been reported that the early presence of beneficial microbes in the gastrointestinal tract can promote resistance to pathogens in broilers by improving the health and integrity of the intestinal tract. These microbes also help to improve the development of the immune system (8). Compared to free-range and litter floor rearing systems, the environment of caged broiler chickens is clean and lacks appropriate microbial stimulation (9, 10). In the absence of appropriate microbial stimulation, the immune system development of animals may be slower, and the immune function may be poor (11). Modern broilers are the products of breeding with the goal of rapid growth. In the process of breeding, the immune function of broilers is a concession for growth performance. Because most of the nutrients in feed are used for muscle growth, fast-growing modern broilers tend to have low disease resistance and high susceptibility to disease. On the other hand, excessive intake of nutrients will put pressure on the digestive system, and the digestion and absorption function of chickens will decline. The excess nutrients in the hindgut will may be improperly fermented by the gut microbiota, and substances such as indole and hydrogen sulfide produced after metabolism can easily cause intestinal inflammation and are not beneficial to intestinal health (12, 13). With the prohibition of antibiotics in feed, health problems among broilers have become more prominent, and the disease incidence of broilers has increased, which has seriously affected the economic benefits of broiler production (14). Therefore, it is necessary to find the key gut microbes related to immune function and target the gut microbiota to enhance the immune function of broiler chickens in cages and improve disease resistance.

Most studies believe that there are differences in the immune function and gut microbiota of animals under different rearing systems. However, most studies on the effects of different rearing systems on the immune function and gut microbiota of animals involve comparisons of outdoor free-range and in-house rearing systems. Previous studies have found that animals that grow in an outdoor environment with more microbes have stronger immune functions (15–17). After concurrent vaccination, compared with indoor captive chickens, free-range and semi-intensive chickens have higher titers against Newcastle disease virus and infectious bronchitis virus in the peripheral blood (18). More microbes and other antigens in the outdoor environment may stimulate the animal’s immune system to develop faster, leading to a stronger immune function state. There were also differences in the gut microbiota of chickens grown indoors and outdoors rearing systems. Compared with indoor caged chickens, outdoor free-range chickens have higher gut microbial alpha diversity (16, 19–21), and the proportions of *Bacteroidetes*, *Actinomycetes*, and *Proteobacteria* were significantly higher (22), as these groups contain more microbes that may be derived from the environment (20, 23). However, the temperature, humidity, light intensity, and rearing density in different rearing systems indoors and outdoors are different, and there are many interference factors that may affect immune function (24–27). Therefore, it is difficult to explain the reasons for the differences in immune function and intestinal microbiota of chickens under different rearing systems, and it is difficult to find a target to improve the immune function of broiler chickens in cage.

Research in the same livestock house can eliminate these interfering factors. The largest antigenic difference between the indoor ground litter and caged broiler breeding environment may be the number of environmental microbes to which they are exposed. However, such studies are rare, and the conclusions are inconsistent. It has been reported that compared with caged layers, the SRBC antibody titer is higher in the serum of ground-floor layer hens (28). Compared with caged chickens, ground litter broilers have higher levels of interleukin-1β (IL-1β) and interferon-γ (IFN-γ) mRNA in the ileum (29). However, some studies have found that there is no significant difference in the number of peripheral blood white blood cells between caged and ground-level laying hens (30). One other study has found the different result. Compared with those of caged chickens, the phagocytic index, the phagocytic activity, and the numbers of peripheral blood eosinophils, lymphocytes, basophils, and monocytes, of ground-level broilers are lower (31). There are relatively few studies have compared the differences in the gut microbiota of poultry grown under the two rearing systems within the same house. In one study it has been shown, compared with the cage group, the ileal microbial alpha diversity and the relative abundance of *Actinomycetes* of broiler chickens in the litter floor group were higher (29). Another study has demonstrated that, compared with the cage group, the cecal microbial alpha diversity of ducks in the litter floor group was higher (32). However, most of the immune function indicators detected in the research of these different rearing systems involved humoral immune function, which is not comprehensive enough, and it is easy to miss the immune function indicators that are sensitive to microbes (28). In addition, most studies on different rearing systems only compare the immune functions of animals and do not perform a correlation analysis between different immune function indicators and different intestinal microbiota (29, 31). Thence, the key microbes that may cause differences in the immune function of chickens with different rearing systems are not yet understood, and it is difficult to provide scientific support for exploring the correlation between the immune function of different rearing systems and intestinal microbiota.

Therefore, this study performed a comparison and correlation analysis of the immune function and gut microbiota of broiler chickens raised in double-layer cages and ground litter floor pens in the same house to identify the key gut microbiota that may be related to the immune function and affect immune system development of broiler chickens. Accordingly, this study provides a theoretical basis for the next step to target gut microbiota, enhance immune function, and improve the intestinal health and disease resistance of broiler chickens.

## RESULTS

### Growth performance and slaughter performance.

**FIG 1:**
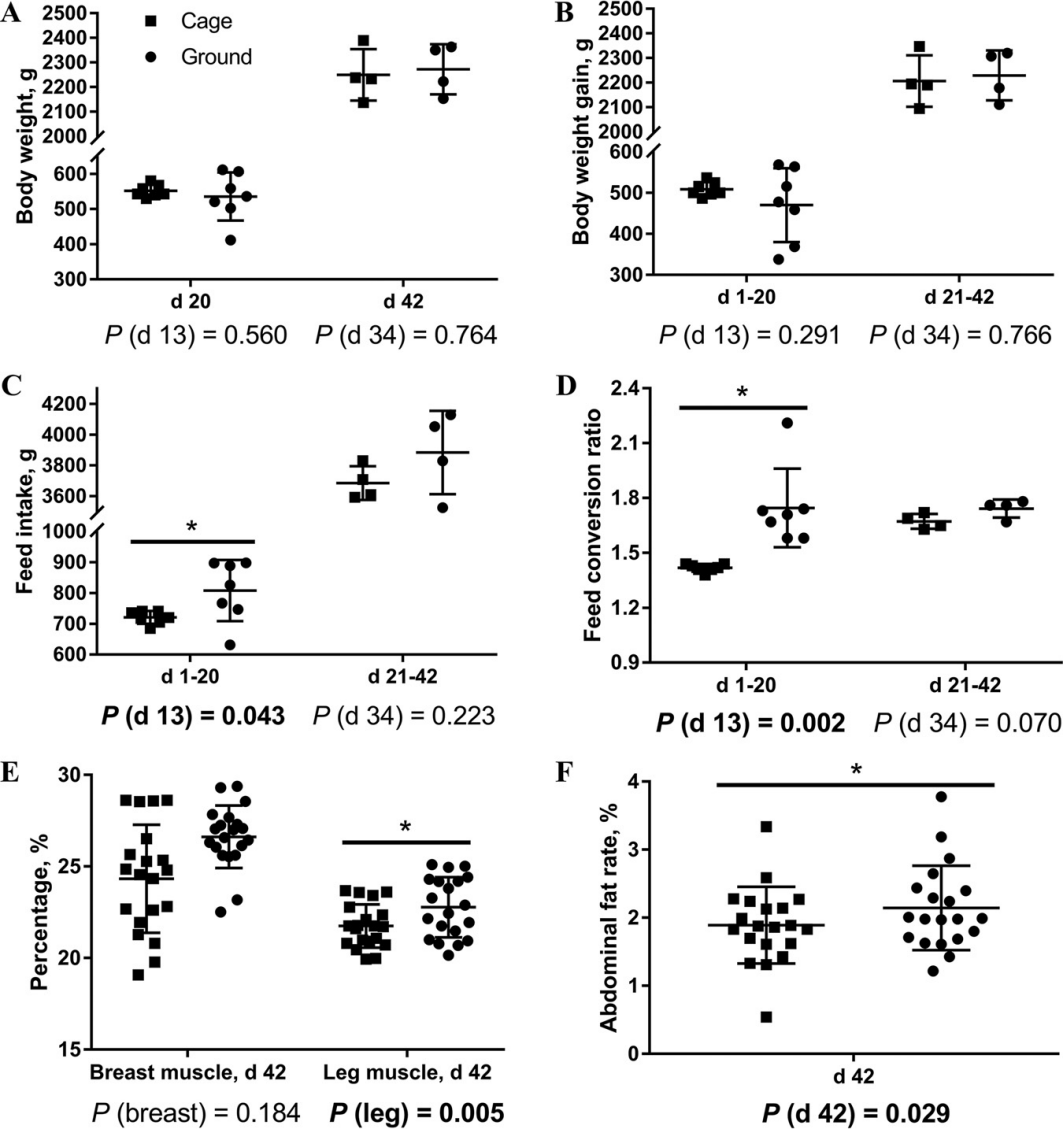
Growth performance and slaughter performance of broiler chickens in cage and ground litter rearing systems. The body weight (A), body weight gain (B), feed intake (C) and feed conversion ratio (D) were analyzed by weighted; d, day. The percentage of breast muscle and leg muscle (E) and abdominal fat rate (F) were analyzed by weighted. All graphs are presented as mean, with SD shown via the whiskers. Statistical differences were determined by one-way ANOVA, with the *P* values for the main effects written out below each plot. *P* values represent the effect of the rearing system. *, *P* < 0.05.

To explore the differences in growth performance between caged and ground litter broilers, we measured feed intake and average body weight of broilers on days 20 and 42. Figure 1A to D and Table 1 show that compared with those of the cage group, the ground litter broilers significantly improved the feed intake (FI) and feed conversion ratio (FCR) in days 1 to 20 (*P < *0.05). The FI during days 1 to 20 increased from 720.71 to 807.86 g, an increase of 12.09%. The FCR during days 1 to 20 increased from 1.42 to 1.75, an increase of 23.24%. There was no significant difference in the average body weight.

**TABLE 1:** Growth performance in cage and ground litter broilers

Items	Cage	Ground	SEM	*P* values^*a*^
BW,^*b*^ g				
Day 20	551.71	535.71	13.008	0.560
Day 42	2,249.78	2,272.69	34.000	0.764
BWG,^*c*^ g				
Days 1–20	508.71	470.43	17.472	0.291
Days 1–42	2,206.88	2,229.51	33.953	0.766
FI,^*d*^ g				
Days 1–20	720.71	807.86	22.090	0.043
Days 1–42	3,685.55	3,883.94	77.294	0.223
FCR^*e*^				
Days 1–20	1.42	1.75	0.060	0.002
Days 1–42	1.67	1.74	0.025	0.070

a *P* values represent the effect of the rearing system.

b BW, body weight.

c BWG, body weight gain.

d FI, feed intake.

e FCR, feed conversion rate.

To explore the difference in slaughter performance between caged and ground litter broilers, we measured and calculated leg, breast, and abdominal fat percentages on day 42. Figure 1E and F and Table 2 show that compared with the rates of the cage group, the leg muscle and abdominal fat rates of broilers in the ground-floor group were higher on day 42 (*P < *0.05). The leg muscle rate grows from 21.75% to 22.78%, an improvement of 4.74%. The abdominal fat rate increased from 1.89% to 2.14%, an increase of 13.23%. There was no difference in the breast rate.

**TABLE 2:** Slaughter performance in cage and ground litter broilers on day 42

Items	Cage	Ground	SEM	*P* values^*a*^
Percentage of breast muscle	24.34	26.62	0.418	0.184
Percentage of leg muscle	21.75	22.78	0.238	0.005
Abdominal fat rate	1.89	2.14	0.095	0.029

a *P* values represent the effect of the rearing system.

### Nonspecific immune function and humoral immune function of peripheral blood.

**FIG 2:**
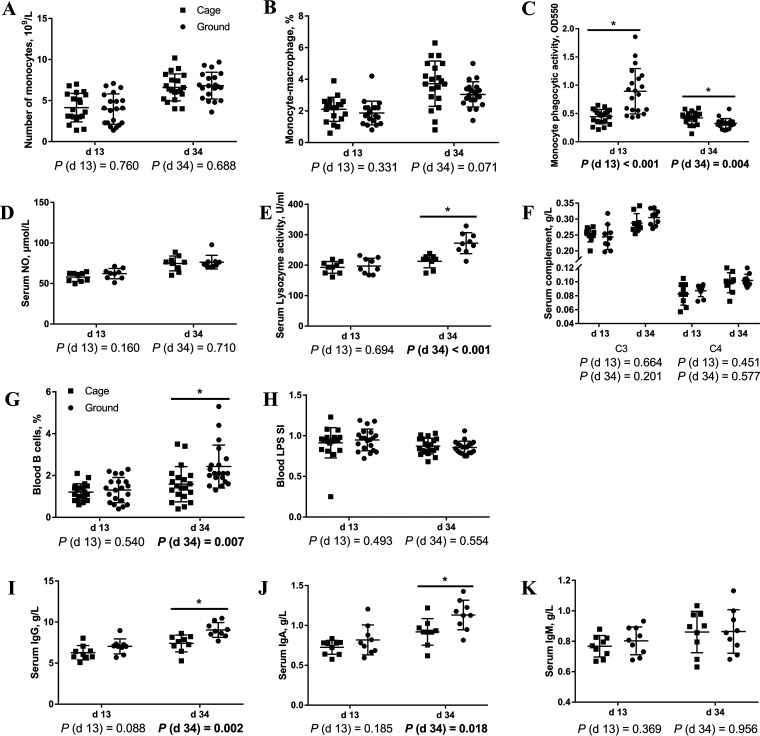
Nonspecific and humoral immune function of peripheral blood of broiler chickens in cage and ground litter rearing systems. The number of monocytes (A) was analyzed by DxH800 blood analyzer (Beckman Coulter Corp., Fullerton, CA, USA). The frequencies of mononuclear/macrophage (B) and B cells (G) of peripheral blood lymphocytes were analyzed by flow cytometry. The phagocytic activity of Monocytes (C) was analyzed by neutral red method. The levels of nitric oxide (NO) (D), lysozyme activity (E), complement (F), IgG (I), IgA (J), and IgM (K) were analyzed by ELISA kit. Peripheral blood lymphocytes were stimulated with lipopolysaccharide (LPS) (H), and the stimulation index (SI) was calculated as described in Materials and Methods. All graphs are presented as mean, with the SD shown via the whiskers. Statistical differences were determined by one-way ANOVA, with the *P* values for the main effects written out below each plot. *P* values represent the effect of the rearing system. *, *P* < 0.05. C3, complement C3; C4, complement C4.

To explore differences in peripheral nonspecific and humoral immune function between caged and ground litter broilers, we examined nonspecific and humoral immune parameters in blood (Fig. 2). Figure 2A to F show that compared with those of the cage group, the day 13 peripheral blood monocyte phagocytic function and day 34 lysozyme activity of broilers in the ground litter group were stronger (*P < *0.05).

As shown in Fig. 2G to K, compared with the levels of the cage group, the levels of peripheral blood B cell percent, serum IgG, and IgA of broilers in the ground-floor group were higher (*P < *0.05). There was a trend toward an increase in the day 13 IgG level (0.05 < *P < *0.1).

### Cellular immune function of peripheral blood.

**FIG 3:**
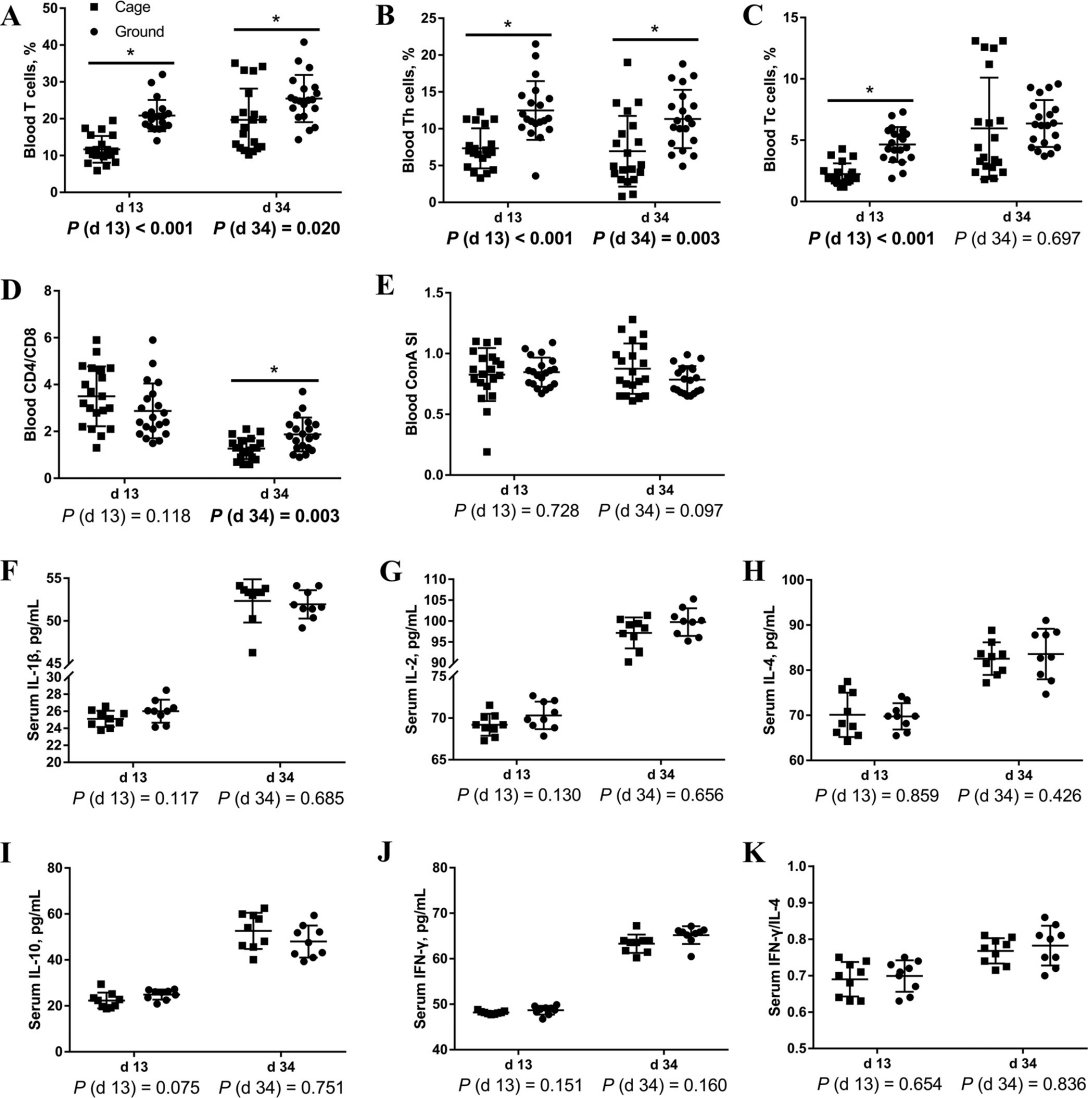
Cellular immune function of peripheral blood of broiler chickens in cage and ground litter rearing systems. The frequencies of T (A), Th (B), and Tc (C) of peripheral blood lymphocytes were analyzed by flow cytometry. The CD4/CD8 (D) of peripheral blood lymphocytes was calculated by dividing the proportion of Th cells by the proportion of Tc cells. Peripheral blood lymphocytes were stimulated with concanavalin A (ConA) (E), and the stimulation index (SI) was calculated as described in Materials and Methods. The levels of IL-1β (F), IL-2 (G), IL-4 (H), IL-10 (I), IFN-γ (J), and IFN-γ/IL-4 (K) in serum were analyzed by ELISA kit. All graphs are presented as mean, with tSD shown via the whiskers. Statistical differences were determined by one-way ANOVA, with the *P* values for the main effects written out below each plot. *P* values represent the effect of the rearing system. *, *P* < 0.05.

In order to explore the differences in peripheral cellular immune function between caged and ground litter broilers, we examined cellular immune parameters in blood (Fig. 3). As shown in Fig. 3, compared with the cage group, the peripheral blood day 13 T cell percent, Th cell percent, and Tc cell percent, and day 34 T cell percent, Th cell percent, and CD4/CD8 percent of the peripheral blood of broilers in the ground flat rearing group were higher (*P < *0.05). There was a tendency toward increased proliferation activity of serum IL-10 on day 13 and peripheral blood T cells on day 34 (0.05 < *P < *0.1).

### Immune organ index and spleen gene expression.

**FIG 4:**
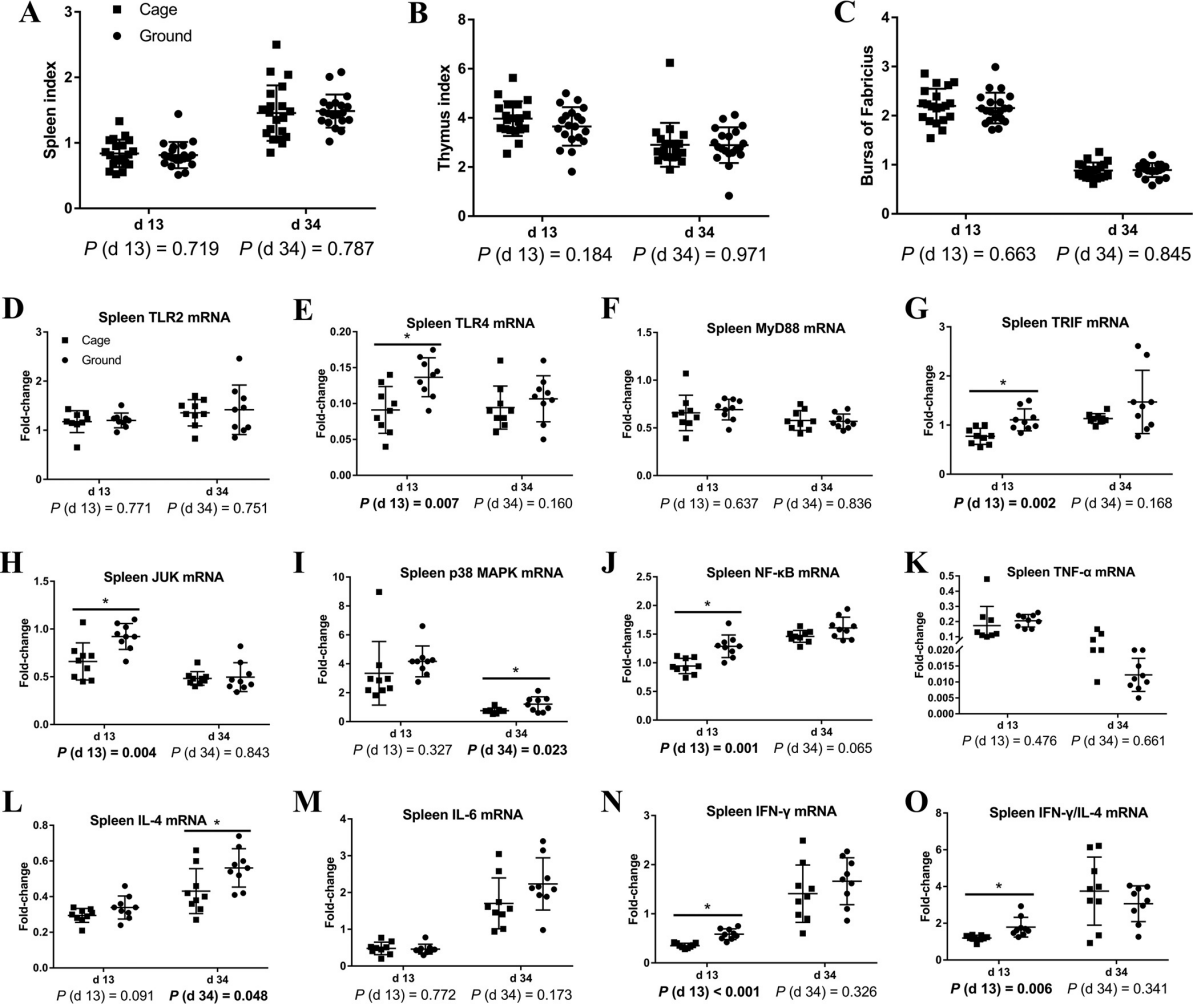
Immune organ index and spleen immune gene expression of broiler chickens in cage and ground litter rearing systems. The spleen index (A), thymus index (B) and bursa of fabricius index (C) were calculated as described in Materials and Methods. The mRNA levels of *TLR2* (D), *TLR4* (E), *MyD88* (F), *TRIF* (G), *JUK* (H), *p38 MAPK* (I), *NF-κB* (J), *TNF-α* (K), *IL-4* (L), *IL-6* (M), *IFN-γ* (N) and *IFN-γ/IL-4* (O) in spleen were analyzed by RT-PCR. All graphs are presented as mean, with the standard deviation (SD) shown via the whiskers. Statistical differences were determined by one-way ANOVA, with the *P* values for the main effects written out below each plot. *P* values represent the effect of the rearing system. *, *P* < 0.05.

For a better understanding of the differences in peripheral cellular immune function in caged and ground litter broilers, we also examined immune-related gene expression in the spleen (Fig. 4). Figure 4 shows that compared with the indices of the cage group, the thymus, bursa, and spleen indices of broilers in the ground-floor group were not significantly different at days 13 and day 34. The spleens of broilers in the ground litter group had higher expression levels of *TLR4* (Toll-like receptor 4), *TRIF*, *NF-κB*, *JUK*, *IFN-γ*, and *IFN-γ/IL-4* on day 13 and *p38 MAPK* and *IL-4* mRNA on day 34 (*P < *0.05). There was a trend toward increased expression levels of *IL-4* mRNA on day 13 and *NF-κB* mRNA on day 34 in the spleen (0.05 < *P < *0.1).

### Intestinal nonspecific immune function and humoral immune function.

**FIG 5:**
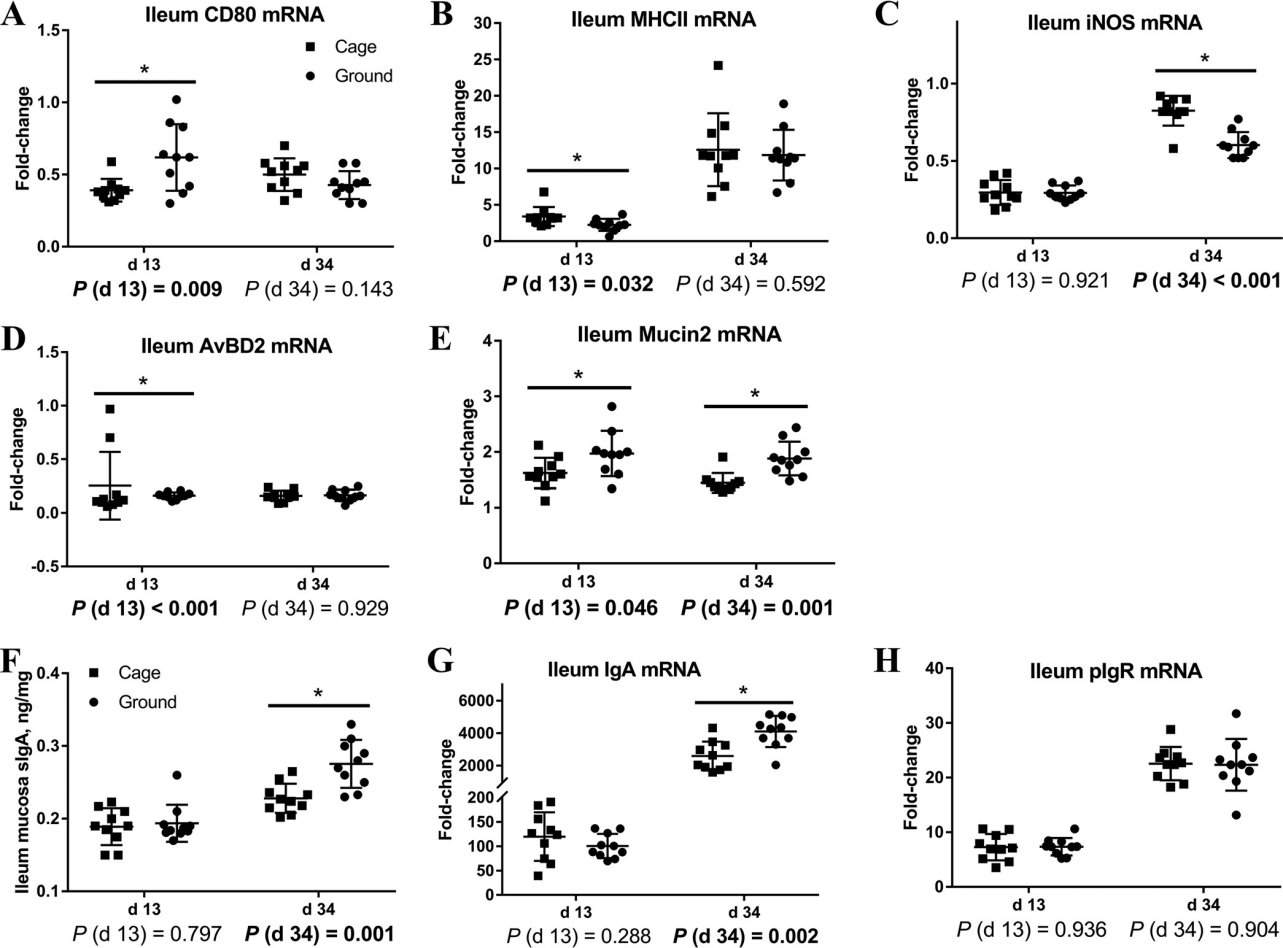
Nonspecific and humoral immune function of ileum of broiler chickens in cage and ground litter rearing systems. The mRNA levels of *CD80* (A), *MHC-II* (B), *iNOS* (C), *AvBD2* (D), *Mucin2* (E), *IgA* (G), and *pIgR* (H) in ileum were analyzed by RT-PCR. The level of sIgA of ileum mucosa (F) was analyzed by ELISA kit. All graphs are presented as mean, with the SD shown via the whiskers. Statistical differences were determined by one-way ANOVA, with the *P* values for the main effects written out below each plot. *P* values represent the effect of the rearing system. *, *P* < 0.05.

In order to clarify the differences in intestinal mucosal nonspecific and humoral immune function between caged and ground litter broilers, we examined nonspecific and humoral immune-related gene expression in the ileum (Fig. 5). Figure 5A to E show that compared with the cage group, the ileum day 13 *CD80*, *AvBD2*, *Mucin2*, and day 34 *Mucin2* mRNA expression levels of broilers in the ground litter group were higher (*P < *0.05). However, compared with the cage group, the ileum day 13 *MHC-II* (major histocompatibility complex II) and day 34 *iNOS* (inducible nitric oxide synthase) mRNA expression levels of broilers in the ground litter group were lower (*P < *0.05).

Figure 5F to H show that compared with expression in the cage group, the ileum day 34 sIgA and *IgA* mRNA expression levels of broilers in the ground litter group were higher (*P < *0.05).

### Intestinal cellular immune function.

**FIG 6:**
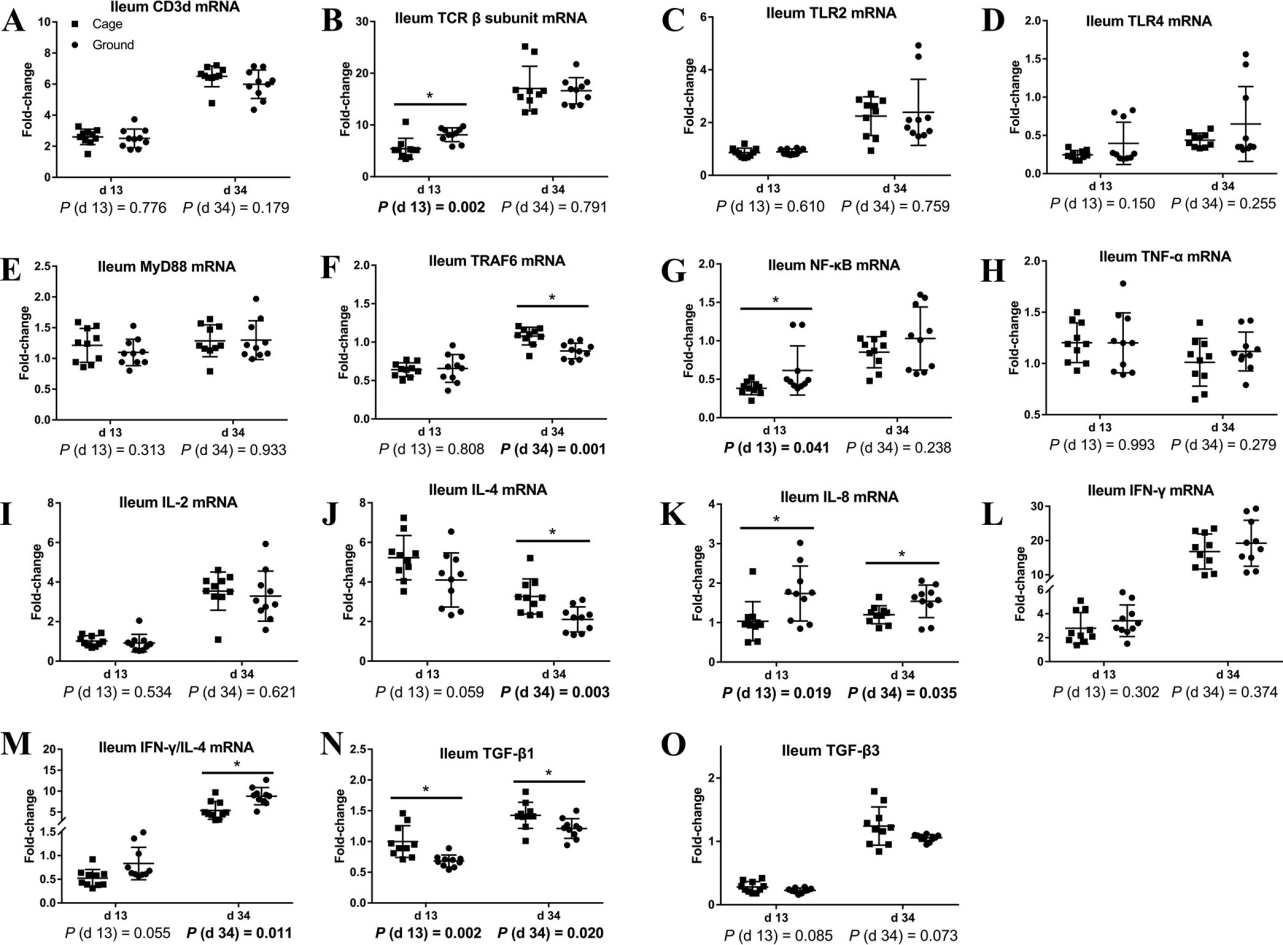
Cellular immune gene expression of ileum of broiler chickens in cage and ground litter rearing systems. The levels of *CD3d* (A), *TCR-β* subunit (B), *TLR2* (C), *TLR4* (D), *MyD88* (E), *TRAF6* (F), *NF-κB*(G), *TNF-α* (H), *IL-2* (I), *IL-4* (J), *IL-8* (K), *IFN-γ* (L), *IFN-γ/IL-4* (M), *TGF-β1* (N), and *TGF-β3* (O) in ileum were analyzed by RT-PCR. All graphs are presented as mean, with the SD shown via the whiskers. Statistical differences were determined by one-way ANOVA, with the *P* values for the main effects written out below each plot. *P* values represent the effect of the rearing system. *, *P* < 0.05.

To clarify the differences in intestinal mucosal nonspecific and humoral immune function between caged and ground litter broilers, we examined the expression of cellular immunity-related genes in the ileum (Fig. 6). As shown in Fig. 6, compared with the levels of the cage group, the ileum day 13 *TCR-β* subunit, *NF-κB*, *IL-8*, and day 34 *IL-8* and *IFN-γ/IL-4* mRNA expression levels of broilers in the ground litter group were higher (*P < *0.05), and the day 13 *TGF-β1* (transforming growth factor-1β) and day 34 *TRAF6* (TNF receptor associated factor 6), *IL-4*, and *TGF-β1* mRNA expression levels were lower (*P < *0.05). There was a trend toward increased ileal day 13 *IFN-γ/IL-4* mRNA expression levels (0.05 < *P < *0.1) and a trend toward decreased mRNA expression levels of *IL-4*, *TGF-β3*, and day 34 *TGF-β3* in the ileum on day 13 (0.05 < *P < *0.1).

### Ileum microbiota.

**FIG 7:**
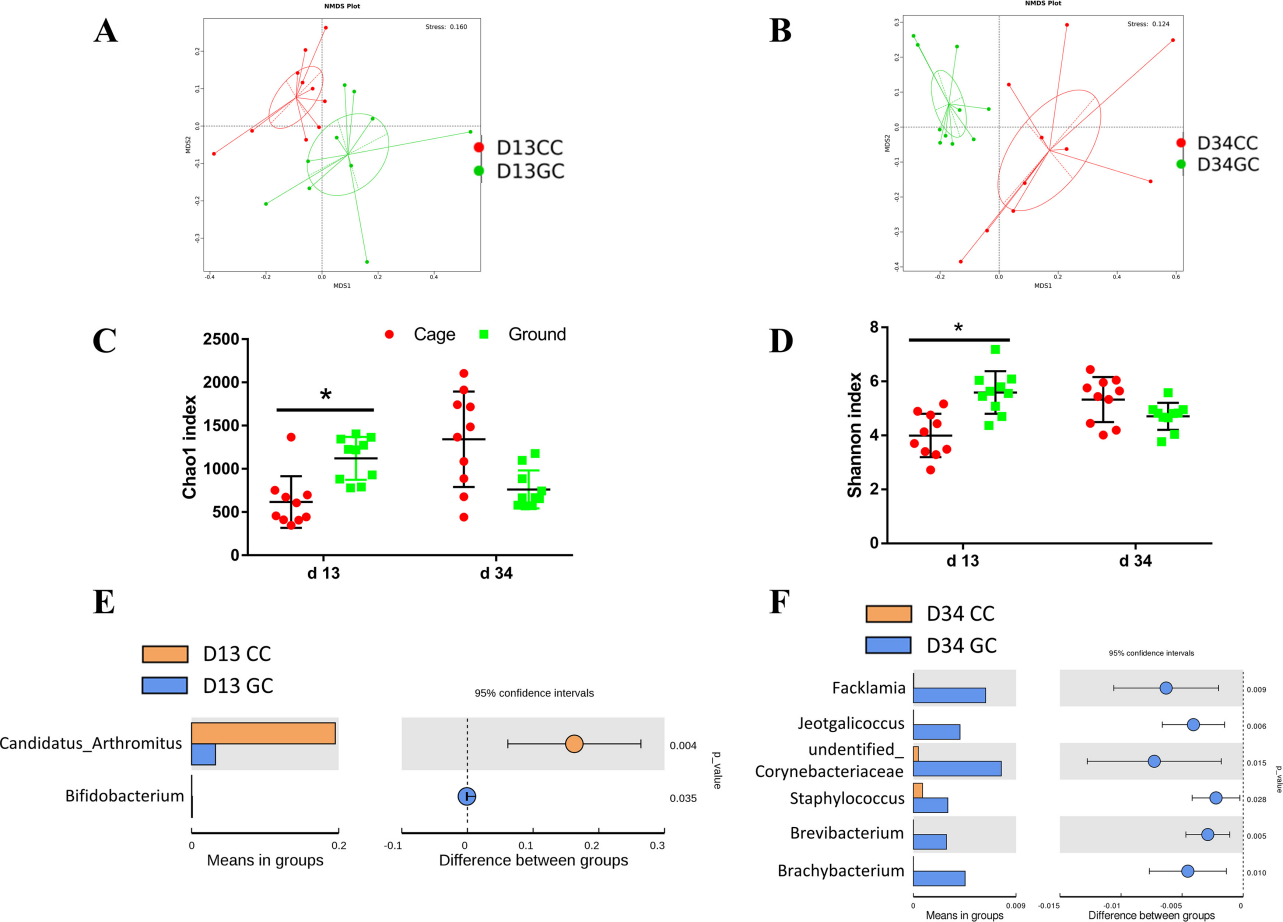
Gut microbiota of broiler chickens in cage and ground litter rearing systems. Beta diversity by nonmetric multidimensional scaling (NMDS) of ileum microbiota of broiler chickens in cage and ground litter rearing systems on day 13 (A) and day 34 (B). Alpha diversity by chao1 (C) and shannon (D) of ileum microbiota of broiler chickens on day 13 and day 34. Differential microbes of ileum of broiler chickens in cage and ground litter rearing systems on day 13 (E) and day 34 (F) on genus level by T-test. CC, cage chickens; GC, ground litter chickens. *, *P < *0.05.

To advance better understanding of the differences in gut microbes between caged and ground-level broilers, we profiled the ileal microbiome by 16S rRNA high-throughput sequencing. From the microbiome data, we calculated the alpha-diversity, beta-diversity of ileal microbiota, and differential microbes at the genus level (Fig. 7).

Figure 7A and B show that the β diversity of the ileal microbiota of the caged and ground-fed broilers was significantly different, and the ileal microbial structure of the two groups at different ages was significantly different.

Figure 7C and D show that compared with the indices of the cage group, the day 13 Shannon index and Chao1 index of ileum microbiota of ground litter group were significantly higher (*P < *0.05).

As shown in Table 3, compared with the abundances in the cage group, the relative abundance levels of day 13 *Bacteroidetes*, *Actinobacteria*, *Proteobacteria*, *Acidobacteria*, *Chloroflexi*, *Gemmatimonadetes*, *Thaumarchaeota*, *Verrucomicrobia*, and day 34 *Firmicutes* in the ileal microbes of broilers in the ground-floor group were higher (*P < *0.05), while the day 13 *Firmicutes* and day 34 *Proteobacteria*, *Bacteroidetes*, *Acidobacteria*, *Chloroflexi*, and *Verrucomicrobia* relative abundance levels were lower (*P < *0.05).

**TABLE 3:** Main *phyla* of ileal microbiota in caged and ground litter broilers

Item	Cage	Ground	SEM	*P* values^*a*^
Day 13				
*Firmicutes*	93.311	75.432	4.193	0.007
*Bacteroidetes*	0.405	4.707	1.510	0.002
*Actinobacteria*	0.081	3.743	1.239	<0.001
*Proteobacteria*	0.280	6.192	1.281	<0.001
*Cyanobacteria*	5.283	8.348	1.257	0.174
*Acidobacteria*	0.007	0.215	0.052	<0.001
*Chloroflexi*	0.009	0.227	0.059	0.001
*Gemmatimonadetes*	0.000	0.055	0.015	<0.001
*Thaumarchaeota*	0.000	0.054	0.015	<0.001
*Verrucomicrobia*	0.062	0.310	0.112	0.015
Day 34				
*Firmicutes*	83.067	95.509	2.129	0.002
*Proteobacteria*	10.311	0.532	1.627	<0.001
*unidentified_Bacteria*	0.737	0.050	0.142	0.003
*Cyanobacteria*	2.272	1.747	0.400	0.069
*Actinobacteria*	0.533	1.666	0.253	0.081
*Bacteroidetes*	2.195	0.359	0.345	0.001
*Acidobacteria*	0.118	0.010	0.014	<0.001
*Tenericutes*	0.032	0.030	0.006	0.820
*Chloroflexi*	0.155	0.015	0.020	<0.001
*Verrucomicrobia*	0.066	0.007	0.012	0.001

a *P* values represent the effect of the rearing system.

Table 4 shows that compared with the abundances in the cage group, the relative abundance levels of day 13 *Enterococcus*, *unidentified_Cyanobacteria*, *unidentified_Corynebacteriaceae*, *unidentified_Enterobacteriaceae*, *Dolosigranulum*, *Peptoniphilus*, Staphylococcus, and *Ulvibacter* in the ileum of broilers in the ground litter group were higher. The relative abundance levels of *Lactobacillus*, Streptococcus, *Facklamia*, *Globicatella*, and *Jeotgalicoccus* in the ileum of day 34 ground litter broilers were higher (*P < *0.05). The relative abundance levels of day 13 *Lactobacillus*, *Candidatus Arthromitus* and day 34 *Romboutsia*, *Enterococcus*, *Phyllobacterium*, *Helicobacter*, and *unidentified_Cyanobacteria* in the ileum of ground litter broilers were lower (*P < *0.05).

**TABLE 4:** Main genera of ileal microbiota in caged and ground litter broilers

Item	Cage	Ground	SEM	*P* values^*a*^
Day 13				
*Lactobacillus*	65.671	53.199	5.273	0.257
*Candidatus_Arthromitus*	19.597	3.254	2.940	0.001
*Enterococcus*	5.525	12.374	2.838	0.650
*unidentified_Cyanobacteria*	5.283	8.326	1.253	0.174
*unidentified_Corynebacteriaceae*	0.001	2.593	1.038	<0.001
*unidentified_Enterobacteriaceae*	0.048	0.174	0.027	0.028
*Dolosigranulum*	0.001	1.389	0.512	<0.001
*Peptoniphilus*	0.000	1.216	0.442	<0.001
Staphylococcus	0.004	1.046	0.354	<0.001
*Ulvibacter*	0.000	0.669	0.297	<0.001
Day 34				
*Lactobacillus*	64.875	86.391	4.252	0.015
*Romboutsia*	5.048	1.062	1.391	0.404
*Enterococcus*	9.323	2.816	1.328	0.023
Streptococcus	1.432	2.257	0.670	0.704
*Phyllobacterium*	2.864	0.109	0.566	<0.001
*Helicobacter*	0.657	0.030	0.135	0.003
*unidentified_Cyanobacteria*	2.264	1.746	0.399	0.069
*Facklamia*	0.003	0.634	0.117	<0.001
*Globicatella*	0.000	0.016	0.003	<0.001
*Jeotgalicoccus*	0.001	0.409	0.072	<0.001

a *P* values represent the effect of the rearing system.

As shown in Fig. 7E and F, compared with the levels in the cage group, the microbes in the ileum of the broiler chickens in the ground-floor group were significantly increased, including *Bifidobacterium* on day 13, *Facklamia*, *Jeotgalicoccus*, *unidentified_Corynebacteriaceae*, Staphylococcus, *Brevibacterium*, and *Brachybacterium* on day 34 (*P < *0.05), while *Candidatus Arthromitus* was significantly reduced (*P < *0.05).

### Correlation heat map.

**FIG 8:**
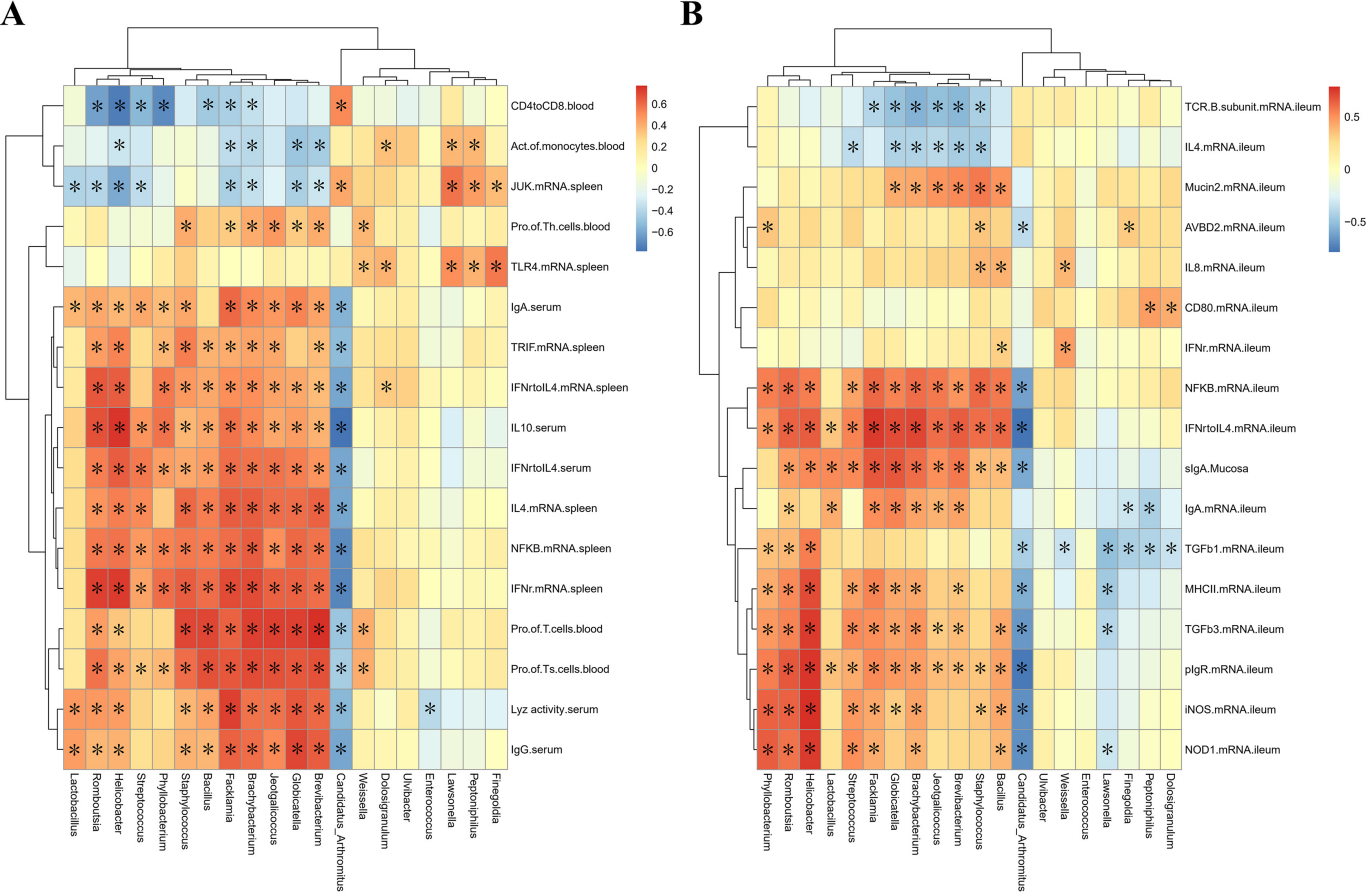
Correlation heat map of significantly different ileum microbes and significantly different immune indexes of broiler chickens in cage and ground litter rearing systems. Spearman’s correlations were calculated for all significantly different peripheral immune parameters (A) and intestinal immune parameters (B) and ileal microbes on genus level. Colors of squares represent *r* values of Spearman’s correlation coefficient. *, *P* < 0.05.

To explore the relationship between the gut microbiota and immune indicators of broiler chickens under different rearing systems, based on the above-mentioned ileal microbiome data, we performed Spearman correlation analysis (Fig. 8) and compared the findings of caged chickens with ground-level broiler chickens. Microbes were associated with a strong immune function state. As shown in Fig. 8A, microbes that were positively correlated with most of the peripheral immune function indicators (serum cytokines, immunoglobulin, spleen gene expression levels, etc.), including *Lactobacillus*, *Romboutsia*, *Helicobacter*, Streptococcus, *Phyllobacterium*, Staphylococcus, *Bacillus*, *Facklamia*, *Brachybacterium*, *Jeotgalicoccus*, *Globicatella*, and *Brevibacterium*, were negatively correlated with peripheral blood CD4/CD8, peripheral blood mononuclear cell phagocytic function, spleen *JUK* mRNA expression. The microorganism negatively correlated with most of the peripheral immune function was *Candidatus Arthromitus*, and the indicators that were positively correlated with *Candidatus Arthromitus* included peripheral blood CD4/CD8 and spleen *JUK* mRNA expression. Microbes that were only positively correlated with a small number of peripheral immune function indicators (peripheral blood mononuclear cell phagocytic function, spleen *JUK*, *TLR4* mRNA expression, and peripheral blood T-cell ratio) were *Weissella*, *Dolosigranulum*, *Lawsonella*, *Peptoniphilus*, and *Finegoldia*. As shown in Fig. 8B, microbes that were positively correlated with most of the intestinal mucosal immune function indicators (ileal mucosal sIgA, ileal immune-related gene mRNA levels) were *Phyllobacterium*, *Romboutsia*, *Helicobacter*, *Lactobacillus*, Streptococcus, *Facklamia*, *Globicatella*, *Brachybacterium*, and *Jeotgalicoccus.* In addition, for *Brevibacterium*, Staphylococcus, and *Bacillus*, the indicators that were negatively related to these bacteria were ileum *TCR-β* subunit and *IL-4* mRNA expression. *Candidatus Arthromitus* was negatively correlated with most of the intestinal mucosal immune function indices. *Weissella*, *Lawsonella*, *Finegoldia*, *Peptoniphilus*, and *Dolosigranulum* were negatively correlated with some intestinal mucosal immune function indices (ileum *TGF-β1*, *IgA*, *MHC-II*, and *TGF-β3* mRNA levels) and negatively correlated with a small number of indices (ileum *AvBD2*, *CD80*, *IFN-γ*, and *IL-8* mRNA levels). The results show that the stronger immune function of ground litter broilers is associated with specifically altered gut microbiota.

## DISCUSSION

To satisfy an increasing demand for dietary protein, the poultry industry has genetically selected broiler breeds for growth performance rather than immune function. The study found that compared with older strains, genetic variants of fast-growing broilers are predicted to enhance growth performance at the expense of immune function (33). At the same time, cage systems have gradually replaced traditional ground litter systems in China. The cage systems realize the separation of chicken and manure and at the same time reduces the stimulation of environmental microbes to broiler chickens during the growth (34). Various reasons may lead to problems such as low immune status, weak immune function, and poor disease resistance in broiler chickens raised in cage systems.

Previous studies have found inconsistent results comparing growth performance of broilers raised in cages and ground litter pens. Some studies have found higher growth performance of chickens raised in ground litter pens. Compared with cage group, broilers raised on floor bedding have better weight gain, but at the same time, the FCR is higher (35). Some studies have found that chickens raised in cages have higher growth performance. Compared with cage group, ground litter group Cobb500 chicks showed lower growth performance (36). Compared with those of the cage group, the deep litter group had higher FCR and lower body weight, body weight gain, FI, and breast muscle rates (37). Other studies have found different results. Researchers also found that there is no significant difference in the growth performance of broiler chickens raised in cages versus floor litter (38). Compared with caged breeding, the weight gain and FCR of free-range Beijing oil chickens were not different, but the FI was lower (39). The results of this study show that cage- and floor-level rearing systems have no effect on the body weight of broilers, and only the FI and FCR of ground-level broilers in the early stage are higher. The high feed intake of broilers raised in the ground litter on days 1 to 10 may be mainly because the pans (days 1 to 3) are placed on the ground litter, and the broilers may throw out feed due to behaviors such as head shaking during the feeding process, resulting in waste of feed and a high FCR. If the pans are replaced by feed troughs placed outside the pens, this effect may be reduced.

Leg muscle, breast muscle, and abdominal fat rates are important indicators of chicken slaughter performance. Our study found that the leg muscle and abdominal fat rates of ground litter broilers were higher, while there was no difference in the breast muscle rate. In this study, although the rearing densities of the two rearing modes were the same, the total area of the ground litter chicken pens was larger than that of the cages, meaning that the broilers can have a greater range of movement. Studies have reported that exercise can increase the percentage of leg and breast muscle (40). We speculate that the ground litter broiler has a larger range of motion, which increases the amount of exercise, thereby increasing the leg muscle rates. This study observed that the lower rate of leg muscles in caged broilers may be due to the small size of the cages and the inability of the broilers to move or even walk a few steps. Therefore, they are more prone to leg diseases than those raised on litter (38). Caged broiler chickens have more gait problems, impaired walking ability, and leg deformities than ground litter broilers do, which may affect the leg muscle rate (41, 42). These results in our study were similar to some previous studies to some extent. Studies have found that compared with indoor pen group, indoor pen with access to a grass paddock group have increased leg and breast muscles (43). Compared with the rates of caged chickens, free-range Beijing oil chickens have a higher abdominal fat rate and a higher breast muscle rate (39). However, some studies had different findings. Studies have found that the rearing system (cage and litter flat rearing) does not affect slaughter performance (35). In a recent study, the breast meat production of litter-raised broilers was higher than that of caged broilers, while the leg meat yield was lower than that of caged broilers (44). Other studies have found that compared with indoor breeding, open-air broilers have increased breast muscles and decreased abdominal fat (45). Many scholars have conducted research on the effects of different rearing systems on chicken slaughter performance, with different conclusions. The differences among the conclusions drawn by studies may result from study design, rearing density, breeds of poultry, temperature, and humidity of rearing systems.

The development and function of the immune system are of great significance to animals, as the immune system helps the body recognize and exclude nonself substances that enter the body, repair damaged tissues, and respond to disease threats. As mentioned above, in view of the possible stress and infection risks in intensive animal husbandry production, it is very important to enhance the immune function of broilers. Due to the short rearing period of commercial broilers (only 42 days), their nonspecific immune function is a very important part of their immune function. The phagocytic capacity of monocytes and nitric oxide, lysozyme, and complement levels are all important indicators of nonspecific immune mechanisms (46–48). This study found that the peripheral nonspecific immune function of ground-raised broilers is stronger and the phagocytic ability and lysozyme activity of peripheral blood mononuclear cells and serum nitric oxide (NO) and C4 levels are significantly improved, which represents the peripheral nonspecific immunity of ground-raised broilers. Previous studies have shown that compared with that of animals living in captivity, animals that grow in a more natural environment have stronger nonspecific peripheral immune functions (49–52). Our research results are similar to previous studies. We speculate that the reason for this similarity may be that more intestinal microbiota in the flat ground environment enter the intestines of broilers, and lipopolysaccharides, peptidoglycans, and their metabolites, which are components of environmental microbiota, pass through the intestines. The proinflammatory cytokines produced by the activated intestinal mucosal immune cells enter the blood, activate dendritic cells (DCs) and macrophages in the peripheral immune organs, and upregulate the nonspecific immune function of the peripheral blood.

During the development of the body’s immune response, peripheral blood lymphocytes participate in multiple processes, and their proliferation and differentiation directly determine the strength of the body’s immune function. T-lymphocyte subsets and the cytokines produced by them play immunomodulatory roles in other immune cells, such as macrophages and B cells, through signal transduction networks (53, 54). This study found that the peripheral cell immune function of ground-level broiler chickens is stronger and the proportion of T cells in peripheral blood and the ratios of Th cells, CD4/CD8, IL-1β, IL-2, and IFN-γ is higher, which represents the level of ground-level broiler chickens. Peripheral cells have stronger immune function and regulate the Th1/Th2 balance toward a Th1 bias. Previous studies have shown that compared with levels in piglets raised outdoors on a farm, piglets raised in an isolator had significantly increased IL-2 produced by mucosal T cells and significantly reduced IL-4 levels (55). Studies have reported that compared with the levels of caged broilers, the levels of *IL-1β* and *IFN-γ* mRNA in the ileum of ground-floor broilers are higher (29), similar to the findings of this study. There are more microbes from the environment in ground-floor broilers, and the proinflammatory cytokines produced by the intestinal mucosal immune cells activated by microbial LPS and peptidoglycan (PGN) and their metabolites can pass the intestinal barrier, enter the blood circulation, stimulate T cells in the peripheral immune organs, increase the proportion of Th cells and the proliferation activity of T cells, increase the levels of the Th1 cytokines IL-2 and IFN-γ, and upregulate the immune function of peripheral blood T cells.

The spleen is an important peripheral immune organ that contains T and B cells. It is related to the final stage of lymphocyte differentiation and the production of memory cells in the secondary immune response and thus plays a key role in immune development (56). This study found that the expression levels of immune-related genes in the spleen of ground-level broilers were higher and the mRNA expression levels of *TLR2*, *TLR4*, *NF-κB*, and the Th2 cytokine *IL-6* were significantly increased. These results indicate that the abundant Gram-positive and Gram-negative microbes in the growth environment may present peptidoglycans and lipopolysaccharides through the digestive tract-blood circulatory system to activate the TLR2/4-NF-κB signaling pathway in the spleen and promote the production of immune cells in the spleen, resulting in the production of more of the Th2-induced cytokine IL-6. The increase in serum IL-6 levels may promote the production of antibodies in peripheral blood (57). Fish studies have found that European eel spleen phagocytes from eels grown in natural rivers have higher potential killing activity than those in captive rearing systems (51), which suggests that in a river environment, eels have strong contact with different pathogens that can activate the natural cellular immune response and are an important line of defense against diseases. Previous studies have reported that compared with that of indoor hens, outdoor hens have a higher spleen index (58). Compared with the levels in ducks reared using an indoor intensive rearing system, TLR7 mRNA expression levels in the bursa, lung, duodenum, ileum, and cecum of outdoor free-range ducks were significantly higher, but there was no difference in *TLR7* mRNA expression in the spleen (59). The difference between spleen TLR expression and the results of this study may be due to the differences in rearing density, TLR functions, and poultry breeds. TLR7 mainly recognizes the receptors of single-stranded RNA viruses, while TLR2 and TLR4 mainly recognize peptide aggregates from the surfaces of microbes, which include saccharides and lipopolysaccharides. The number of microbes that ground litter broiler chickens are exposed to during the growth process is greater, and TLR expression in spleen immune cells increases in response.

The immunoglobulins IgG, IgM, and IgA are produced at the highest levels in the peripheral blood of poultry and are important indicators of the functional status of the humoral immune system (60). This study found that the peripheral humoral immune function of ground-level broilers was stronger, and the serum IgG and IgA levels were higher. Previous studies reported that compared with titers of caged layer hens, SRBC antibody titers in the serum of ground-level layer hens were higher (28). Compared with titers of indoor captive chickens, free-range and semi-intensive chickens have higher titers against Newcastle disease virus and infectious bronchitis virus in peripheral blood (18). Fish studies have found that European eels grown in natural rivers have higher serum Ig levels than those of eels from captive rearing systems (51). Our research results are similar to those reported in previous studies. When animals grow in an environment with more microbes, environmental microbes may enter the intestine and colonize. Intestinal mucosal immune cells are activated by LPS and PGN on the surfaces of these microbes and the metabolites produced by bacteria. Proinflammatory cytokines produced by immune cells enter the blood circulation through the intestinal barrier, thereby stimulating immune cells in peripheral immune organs. Immune cells release more of the Th2 cytokine IL-6, induce B cell activation, increase IgG and IgA levels, and enhance the peripheral humoral immune function of broilers.

The intestine is not only the main digestive organ of animals but is also an important immune organ. The intestine contains a large number of immune cells, such as intestinal intraepithelial lymphocytes and lamina propria lymphocytes. These immune cells are mainly composed of T cells, B cells, and macrophages (61). Macrophages produce NO after being stimulated by bacterial pathogen-related molecular patterns and IFN-γ. CD80 is a marker of macrophage activation (62). Mucin2 is mainly secreted by intestinal goblet cells, which participate in the formation of the intestinal barrier, prevent the translocation of intestinal bacteria, and maintain intestinal homeostasis (63). This study found that the nonspecific immune function of the intestinal mucosa of broilers in the ground-level broiler group was stronger, which was manifested by higher mRNA expression levels of *CD80* and *Mucin2* in the ileum. A previous study found that compared with the cage group, ground litter Beijing You chickens had higher mRNA level of Mucin2 in the jejunum (64). Compared with piglets raised in an isolator, four farm piglets had a flora at 2 and 5 days that was comparable to older animals and mucosal DCs that were also increased significantly (55). The findings of these studies were similar to our study. Other studies have found that microbes can stimulate the maturation of DC cells and goblet cells (65, 66). The stronger immune recognition ability of intestinal DC cells and the stronger ability of goblet cells to secrete Mucin2 in ground litter broilers may be caused by the stimulation of immune cells by microbes entering the intestine from the environment.

CD3d and TCR are surface receptors and cell markers of T cells, and these proteins are essential for T cells to function (67). Th cells are an important subgroup of T cells that are mainly related to Th1 and Th2 immune responses. IFN-γ and tumor necrosis factor α (TNF-α) secreted by intestinal Th1 cells can downregulate the secretion of IgA (68). IL-4 and IL-10 and other cytokines produced by Th2 cells can induce and enhance the expression of the secreted component of IgA (69). This study found that the intestinal cellular immune function of broilers in the ground litter group was stronger, and the mRNA expression levels of ileum *TCR-β* subunit, *TLR2*, *TLR4*, *IL-8*, *IFN-γ*, and *IFN-γ/IL-4* were higher, while the mRNA expression levels of *IL-4*, *TGF-β1*, and *TGF-β3* were lower. These findings indicate that the Th1/Th2 cytokines produced in the intestine of ground-level broilers are biased toward Th1, the level of anti-inflammatory cytokines is lower, and the immune function of intestinal cells is stronger. Previous studies reported that compared with those of caged broilers, the mRNA expression levels of *IL-1β* and *IFN-γ* in the ileum of ground-floor broilers were higher (29). Compared with piglets raised outdoors on the farm, piglets raised in an isolator had significantly increased IL-2 produced by mucosal T cells and significantly reduced IL-4 (55). Compared with the piglets in the isolator, the outdoor farming environment significantly reduced the number of CD25^+^/Foxp3+ regulatory T cells in the proximal and distal jejunum of the piglets at 28 days of weaning (70). Compared with chicks raised on new litter, litter recovered after three uses was associated with significantly increased *IL-1* mRNA levels in cecal tonsils at days 10 and 35 and a significantly reduced percentage of cecal tonsil CD4^+^/CD25^+^ cells. The intestinal immune response of birds raised on regenerated bedding tends to be inflammatory, while fresh bedding treatment tends to be anti-inflammatory (71). In general, the results of previous studies were similar to these study results. The intestines of animals raised on the ground litter, outdoors, and in the wild are closer to a stronger immune state similar to mild inflammation, and the Th1/Th2 balance is upregulated. This may be because bacteria in more complex environments, such as the wild, enter the intestine, which in turn activates the immune-related signaling pathways of TLRs on immune cells and activates immune cells such as dendritic cells. Activated immune cells produce more proinflammatory factors, leaving the animal’s intestines in a state prone to mild inflammation.

Intestinal mucosal sIgA occupies a dominant position in mucosal immunity and is the first line of defense to protect intestinal epithelial cells from intestinal toxins and pathogenic microbes. It prevents pathogens from adsorbing and entering epithelial cells, traps them in mucus, and passes through mucus and fibers to remove bacteria (72). This study found that the intestinal mucosal humoral immune function of broiler chickens in the ground flat rearing group was stronger, and the mRNA expression levels of *IgA* and *pIgR* in the ileum were higher. Previous studies reported that compared with the titers in caged layer hens, SRBC antibody titers in the serum of ground-level layer hens were higher (28). There have been few studies on the effects of different rearing systems in the same chicken house on the intestinal humoral immune function of chickens. We speculate that more environmental microbiota in the growth environment of ground litter broilers enter the intestine and directly bind to immune cell TLR receptors through LPS and PGN or bind to Th-cell receptors through bacterial metabolites such as short-chain fatty acids (SCFAs) and bile acids. Activated Th2 cells produce more Th2 cytokines, activate B cells, upregulate IgA and pIgR mRNA transcription levels, and ultimately improve intestinal humoral immune function (5, 73–75).

Previous studies have shown that there is an inseparable relationship between animal immune function and gut microbiota. The interaction between the immune function of broilers and microbes may depend on the production of a variety of biologically active small molecule metabolites. Intestinal microbiota activates immune cells through lipopolysaccharide and peptidoglycan (76) and the production of small molecule active metabolites (short-chain fatty acids, secondary bile acids, and indole) (77). The proinflammatory cytokines produced by activated intestinal immune cells stimulate peripheral immune organs and regulate the peripheral immune system after circulating in the blood.

The intestine of broilers has a very complex microbiota. The intestinal microbiota plays an important role in nutrient digestion and absorption, regulating the immune system, preventing diseases, and maintaining physiological functions (78, 79). The diversity and composition of the intestinal microbiota of broilers are regulated by many factors, such as diet, age, antibiotics, genetics, immune response, and pathogen infection (80).

It is generally believed that the higher the species abundance is and the higher the gut microbial diversity is, the better, which is more conducive to the stability of the gut microbial composition. This study found that the ileum microbial alpha diversity of ground-floor broilers is higher and that the microbiota is more diverse, which can improve the body’s homeostasis, promote the digestion and absorption of nutrients, and resist pathogens (81). Previous studies have found that in the same chicken house, compared with the index of caged broilers, the ileal microbial Shannon index of ground litter broilers is higher (29). Studies have also found that compared with ground-floor breeding, broiler chickens raised outdoors have a higher alpha diversity of cecal microbiota (23). Compared with the diversity of caged broilers, the cecal microbial alpha diversity of broilers raised outdoors is higher (20). When laying hens are raised in a free-range environment, they are exposed to a large number of environmental microbes, which enrich their gut microbiota during the process of laying hens pecking on litter, scratching, and dust bathing (9, 10). These studies are similar to our research results. The reason for the higher ileal microbial alpha diversity in stocking and ground litter broilers may be the greater variety and number of microbes in the rearing environment. Broilers come into contact with these microbes when they eat and peck on litter, and then the bacteria colonize the intestines.

This study found that potentially harmful bacteria (Streptococcus, Staphylococcus, *Brachybacterium*, *Brevibacterium*, *Dolosigranulum*, and *Peptoniphilus*) and litter breeding bacteria (*Facklamia*, *Globicatella*, and *Jeotgalicoccus*) significantly increased in the ileum microbes of ground-floor broilers and significantly reduced *Candidatus Arthromitus*. In our study, through Spearman correlation analysis, we found that Streptococcus, Staphylococcus, *Helicobacter*, *Brachybacterium*, *Brevibacterium*, *Facklamia*, *Globicatella*, and *Jeotgalicoccus* were significantly related to peripheral and intestinal mucosal immune indicators in the ileal microbiota of ground-floor broilers. Among them, Streptococcus and Staphylococcus are potential pathogens (82, 83). After entering the intestine of broiler chickens, they may act as antigens to cause immune stress and can lead to the upregulation of immune function and inflammation in broiler chickens. Other studies have shown that *Helicobacter* in the mouse colon is positively correlated with the percentage of peripheral blood mononuclear cells, the monocyte percent, and liver alanine aminotransferase and is negatively correlated with serum TNF-α levels and the thymus index (84). These results are similar to the results of our research. Potential pathogenic bacteria may act as antigens to activate immune cells and improve immune function after entering the intestinal tract of broilers.

*Facklamia*, *Globicatella*, and *Jeotgalicoccus* are Gram-positive cocci that are often found in the litter and air of chicken houses. *Facklamia* is one of the most abundant bacteria in litter (85). *Jeotgalicoccus* is found in pig nose swabs, and the environment carries the plasmid of the multidrug resistance gene cfr (86). Our research has found that *Facklamia*, *Globicatella*, and *Jeotgalicoccus* are more present in the ileum of ground-floor broilers. This finding suggests that the three types of bacteria in the ileum of ground-floor broiler chickens may be obtained from litter and that the litter model of ground-floor broilers may have a higher risk of lateral spread of antibiotic resistance genes. More interestingly, in our research, through Spearman correlation analysis, we found that *Facklamia*, *Globicatella* and *Jeotgalicoccus* were positively correlated with many immune indicators, and these three genera are common bacteria in litter (85). This result suggests that these three genera may be the key bacteria that regulate the immune function of ground-floor broilers, and their source can be litter. Perhaps exposing hatched caged broilers to litter containing these three microbes may have a good effect on the development of the immune system.

Intestinal microbiota mainly regulates the host’s intestinal mucosal immune function through *de novo* synthesis of lipopolysaccharides and peptidoglycans and the use of host diets to produce small molecule active metabolites SCFAs, secondary bile acids, and indole. These substances stimulate immune cells in the peripheral immune system to regulate peripheral immune function after circulating in the blood. Our research found that the bacteria that significantly increased the ileum microbiota of ground-floor broilers were mainly Gram-positive bacteria, and the peptidoglycan and lipopolysaccharide on the surface of the bacteria might affect the host’s immune function. This result suggests that because our caged broilers grow in a cleaner environment, there are fewer microbial antigens from the natural growth environment, which may be the reason for the weaker immune status of caged broilers and their low immune function and disease resistance.

### Conclusion.

In summary, compared with those of caged broilers, ground litter broilers have stronger immune function, higher ileal microbial alpha diversity, more opportunistic pathogens and litter breeding bacteria, and more microbes to stimulate the immune system of the broilers. We also found that opportunistic pathogens and litter breeding bacteria are strongly related to the immune function of broilers, and litter breeding bacteria may be used as potential probiotic additives, with future immune enhancement effects. We confirmed the differences in microbial gut composition and immune function of broiler chickens in the two rearing systems. This work provides new data for broiler chickens raised with different rearing systems and increases the understanding of the interaction between broiler gut microbiota and immunity.

## MATERIALS AND METHODS

### Ethics statement.

The experimental animal procedures were approved by the China Agricultural University Animal Care and Use Committee (Beijing, China).

### Experimental animals, rearing systems, and diets.

Eighty male Arbor Acres broilers were selected, euthanized, and sampled after anesthesia at days 1, 13, and 34. Except for 6 replicates at day 1, each treatment group had 20 replicates at other days, and all groups had *ad libitum* access to food and water. According to the recommendations of the National Research Council (NRC 2012), drug-free corn-soybean meal diets were formulated to meet or exceed the nutritional requirements of broilers. Please refer to Table 5 for the specific diet formula. Standard management procedures were used throughout the experiment. The chicken cages were 100 cm in length × 70 cm in width × 40 cm in height, and the rearing density was 14.3 birds/m^2^. The ground chicken pens were 310 cm in length × 60 cm in width × 75 cm in height, and the rearing density was 14.4 birds/m^2^. According to the actual litter floor broilers breeding situation, we used rice husks as bedding litter and stacked the rice husks to 8 cm in height until the end of the experiment. Room temperature was maintained at 33°C during first 5 days and then gradually decreased by 2°C weekly until a final room temperature of 24°C was reached. Artificial light was provided in a 23-h light:1-h dark program. Double-layer cages and ground litter floor pens were located in the same chicken house and had the same levels of light, temperature and humidity. Samples were collected at days 1, 13, and 34 to determine indicators, and the test period was 42 days.

**TABLE 5:** Ingredients and composition (calculated and analyzed nutrients) of the experimental diets^*a*^

Item	Days 1–20	Days 21–42
Composition, %		
Corn (7.8% CP)	51.38	60.02
Soybean meal (46% CP)	40.71	25.54
Corn protein flour	0.00	5.66
Soybean oil	3.75	3.32
Wheat flour	0.00	2.00
CaHPO_3_·2H_2_O	1.86	1.33
Stone powder (37%)	1.24	1.14
Sodium chloride	0.35	0.35
dl-methionine (98%)	0.20	0.070
l-lysine HCL (98%)	0.00	0.19
Vitamin premix^*b*^	0.03	0.03
Mineral premix^*c*^	0.20	0.20
Choline chloride (50%)	0.25	0.16
Sandoquin (ethoxyquinoline)	0.030	0.00
Calculated nutrient levels^*d*^		
Metabolizable energy (Kcal/kg)	2,928.97	3,100.00
Crude protein	21.76	20.00
Calcium	1.01	0.90
Available phosphorus	0.44	0.35
Lysine	1.14	1.00
Methionine	0.54	0.40

a Diets were in mash form (%, unless otherwise noted, as-fed basis). CP, crude protein.

b Vitamin premix provided per kg of complete diet: vitamin A (retinylacetate), 9,500 IU; vitamin D3 (cholecalciferol), 2,500 IU; vitamin E (dl-a-tocopherol acetate), 30 IU; vitamin K3 (menadione sodium bisulfate), 2.65 mg; vitamin B12 (cyanocobalamin), 0.025 mg; biotin, 0.30 mg; folic acid, 1.25 mg; nicotinic acid, 50 mg; d-pantothenic acid, 12 mg; pyridoxine hydrochloride, 6.0 mg; riboflavin, 6.5 mg; thiamine mononitrate, 3.0 mg.

c Mineral premix provided per kg of complete diet: iron, 80 mg; copper, 8 mg; manganese, 100 mg; zinc, 80 mg; iodine, 0.35 mg; selenium, 0.15 mg.

d Calculated value based on the analysis of experimental diets.

### Growth performance.

Body weight and feed consumption were determined at days 20 and 42. Weight gain, feed intake, and FCR were calculated for starter, grower, and overall periods. Broiler chickens were checked every day, and any chicken that died or was removed was weighed and used to correct for the feed intake and FCR.

### Slaughter performance.

We measured single breast meat (including pectoralis major and pectoralis minor), single leg muscle (including thigh and drumstick) and abdominal fat. The weight percentages of breast meat, leg meat, and abdominal fat to body weight were calculated.

### Immune organ index.

At days 13 and 34, 10 healthy chickens were randomly selected for each treatment (1 for each replicate). After weighing, the chickens were euthanized by intravenous injection of 50 mg/kg body weight sodium pentobarbital solution under the wings. The thymus, spleen, bursa of fabricius, and liver were collected and weighed. The immune organ index was calculated with the following formula: immune organ index (g/kg) = immune organ weight (g)/body weight (kg).

### Peripheral blood mononuclear cell isolation.

The isolation of peripheral blood mononuclear cells (PBMCs) was conducted as previously described (87) using density gradient centrifugation with Ficoll-Paque Plus following the manufacturer’s guidelines. Briefly, six healthy chickens (1 bird per replicate) were randomly selected from each treatment group on days 13 and 34. The blood samples were collected in tubes coated with heparin from the wing vein and then diluted 1:1 with sterile calcium- and magnesium-free Hanks’ balanced salt solution (CMF-HBSS; Sigma). The diluted samples were placed on ice and then carefully layered into a tube containing an equal volume of Ficoll lymphocyte separation medium (Histopague-1077, Tianjin HaoYang Biological Manufacture Co., Ltd., China) to form a distinct layer above the Ficoll. Following centrifugation at 400 × *g* for 30 min at room temperature, the white flocculent material on the interface between the plasma and the lymphocyte separation medium was transferred to a clean tube using a sterile transfer pipette. The lymphocyte suspension was washed three times with RPMI 1640 (Invitrogen Corp., Grand Island, NY, USA) incomplete culture medium and then resuspended in 2 mL of RPMI 1640 complete culture medium supplemented with 5% (vol/vol) fetal calf serum, 0.5% penicillin (final concentration, 100 U/mL), 0.5% streptomycin (final concentration, 100 μg/mL), and 1% HEPES (final concentration, 24 mmol/L; Amresco 0511; Amresco Inc., Cleveland, OH). The live cells were detected using the Trypan blue dye exclusion technique and a microscope (DM6000B; Leica Microsystems, Wetzla, Germany). The cell suspensions were diluted to a final concentration of 1 × 10^7^ cells/mL in RPMI 1640 medium for subsequent analysis.

### Peripheral blood mononuclear cell proliferation.

A 3-(4,5-dimethylthiazol)-2,5-diphenyltetrazolium bromide (MTT; Sigma Chemical Co., St. Louis, MO) assay was used to determine the peripheral blood and ileum lymphocyte proliferation response. Briefly, 100 μL of the PBMC suspension and 100 μL of RPMI 1640 in the absence or presence of 90 μg/mL concanavalin A (ConA; C2613, Sigma Chemical Co.) or 50 μg/mL lipopolysaccharide (L3129; Sigma Chemical Co.) were added to a 96-well microtiter plate (Costar 3599; Corning, Inc., Corning, NY). The cultures were set up in triplicate. After a 68-h incubation in a 5% CO_2_ incubator (MCO-18AIC CO_2_ incubator; Sanyo Electric Biomedical Co., Ltd., Tokyo, Japan) at 39°C, MTT was added to each well at a final concentration of 5 mg/mL. The cells were incubated for an additional 4 h, and then, 100 μL of 10% sodium dodecyl sulfate dissolved in 0.04 mol/L HCl solution was added to each well to lyse the cells and solubilize the MTT crystals. Finally, the absorbance value of each sample was determined using an automated ELISA reader (model 550 Microplate Reader; Bio-Rad Pacific Ltd., Hong Kong, China) at 570 nm. The stimulation index (SI) for each sample was calculated based on the following formula: SI = (absorbance value of mitogen stimulated cells)/(absorbance value of media without mitogen).

### Phagocytic activity of mononuclear lymphocytes in peripheral blood.

The phagocytic activity levels of peripheral blood mononuclear lymphocytes were measured by the neutral red assay method. In short, peripheral blood PBMCs were collected, and the number of cells was adjusted to 2 × 10^6^/mL with RPMI 1640 medium. One hundred microliters was then incubated in a 96-well cell culture plate for 2 h (with 3 replicate wells), the supernatant was discarded, 200 μL/well of 0.1% neutral red solution (N299163, Shanghai Aladdin Biochemical Technology Co., Ltd., China) was added, and the cells were further incubated for 2 h. The supernatant was then discarded, and any remaining neutral red was washed away with PBS (3 times). Cell lysate was added at 200 μL/well (ethanol:acetic acid = 1:1) and kept in the dark at 4°C for 12 h, and the optical density (OD) value was measured at 550 nm.

### Determination of T-cell subsets, B cells, and monocytes/macrophages in peripheral blood PBMCs by flow cytometry.

The percentages of cluster of differentiation three receptors CD3^+^, CD4^+^, CD8^+^, Bu-1+, and monocyte/macrophage cells in the peripheral blood mononuclear cell samples were analyzed by flow cytometry as previously described (88, 89). Briefly, the following primary monoclonal antibodies were diluted in PBS (pH 7.2): IgG1κ mouse anti-chicken-CD3-APC-labeled antibody (8200-11), IgG1κ mouse anti-chicken-CD4-Alexa Fluor 700-labeled antibody (8210-27), IgG1κ mouse anti-chicken-CD8-Pacific Blue-labeled antibody (8220-26), IgG1κ mouse anti-chicken-Bu-1-FITC-labeled antibody (8395-02), and IgG1κ mouse anti-chicken-monocyte/macrophage-PE-labeled antibody (8420-09) (Southern Biotechnology Associates Inc., Birmingham, AL). A volume of 100 μL of PBMCs (2 × 10^6^ cells) was added into a 1-mL Eppendorf tube; the contents of the tube were stained with 25 μL of diluted primary monoclonal antibody (1:100 dilution) and the negative isotype control IgG (mouse IgG1κ-APC, mouse IgG1κ-Alexa Fluor 700, mouse IgG1κ-Pacific Blue, mouse IgG1κ- FITC, and mouse IgG1κ-PE). After incubation for 45 min at room temperature, the cells were washed twice with cold PBS and centrifuged for 30 min at 1,800 × *g* to remove the unbound primary antibodies. A total of 300 μL of hemolysin solution diluted in PBS (1:25) was added to each tube. Finally, the cells were washed twice and adjusted to a final volume of 500 μL. Five-color flow cytometric analysis was conducted using a Navios EX flow cytometer with 10 colors (Beckman Coulter, Corp., Fullerton, CA, USA) at Xi-Yuan Traditional Chinese Medicine Hospital, Chinese Academy of Medicine Science, China. The percentages of CD3^+^ T cells, CD3+CD4^+^ T cells, CD3+CD8^+^ T cells, B cells, and monocytes/macrophages were subsequently calculated.

### Serum NO, lysozyme activity, cytokine, immunoglobulin, and mucosal sIgA levels.

The levels of IL-1β, IL-4, IL-10, and IFN-γ in the serum were determined using commercial ELISA kits (Genorise Scientific Inc., Paoli, PA, USA). According to the instructions, a chicken IgG ELISA kit (E30-104, Bethyl Laboratories Inc., Montgomery, TX, USA) was used to determine the level of IgG in the serum. Serum IgG, IgA and IgM levels were determined using a commercial ELISA kit (IDEXX Laboratories Inc., Westbrook, ME, USA) according to the protocol recommended by the manufacturer. Serum NO and lysozyme activities were measured using commercial ELISA kits (A013-2-1 and A050-1-1; Nanjing Jiancheng Institute of Bioengineering, Nanjing, China). Ileal mucosa sIgA was measured using a commercial ELISA kit (YM-SQ2632; Shanghai Yuan Mu Biotechnology Co., Ltd., Shanghai, China).

### Determination of gene expression in spleen and ileum by RT-PCR.

On days 1, 13, and 34, spleen and ileum molecular samples were quickly frozen in liquid nitrogen and transferred to a −80°C freezer to measure the expression of spleen and ileum immune function-related genes. The total RNA of the spleen and ileum was extracted by the TRIzol reagent method (15596018; Invitrogen Life Technologies, Breda, The Netherlands). The quality and quantity of the total RNA were measured with a spectrophotometer (NanoDrop-2000; Thermo Fisher Scientific, Waltham, MA, USA) using the 260- to 280-nm absorbance ratio. First-strand cDNA was synthesized using a PrimeScript RT reagent kit with gDNA Eraser (Perfect Real Time; TaKaRa Biotechnology Co. Ltd., Dalian, China) according to the manufacturer’s instructions. The cDNA was used to perform quantitative real-time PCR (Applied Biosystems 7500 Fast real-time PCR system, USA) for target-gene expression according to the standard protocol (90). Primer sequences (Table 6) for chicken were designed and synthesized by Sango Biotech Co., Ltd (Shanghai, China). Using GAPDH as an internal reference, the results are shown as 2^−△△CT^. The mRNA levels of target genes in the spleen and ileum of 1-day-old broiler chickens were set to 1, to calculate the relative fold changes in the mRNA levels of the same target genes at following day of ages.

**TABLE 6:** Sequences of the oligonucleotide primers used for quantitative real-time PCR^*a*^

Gene^*b*^	Primer sequence^*c*^ (5′ to 3′)	GenBank accession no.
*GAPDH*	F: AGAACATCATCCCAGCGTCC	NM_204305
	R: CGGCAGGTCAGGTCAACAAC	
*CD80*	F: CTGTTCCTTCACATCCTGAGAG	Y08823
	R: CTTCAACACCATCTATTTGCCAG	
*MHCII*	F: CCACGGACGTGATGCAGAAC	113206149
	R: ACCGCGCAGGAACACGAAGA	
*iNOS*	F: GAACAGCCAGCTCATCCGATA	U34045
	R: CCCAAGCTCAATGCACAACTT	
*AvBD2*	F: TTCCGTTCCTGCTGCAAATG	NM_204992
	R: GCCTGGAAGAAATTTTCAAAGCTC	
*Mucin2*	F: TTCATGATGCCTGCTCTTGTG	XM_421035
	R: CCTGAGCCTTGGTACATTCTTGT	
*Lyz C*	F: GACGATGTGAGCTGGCAG	NM_205281
	R: GGATGTTGCACAGGTTCC	
*pIgR*	F: ATTTGTCACCACCACAGCCA	NM_001044644
	R: GAGTAGGCGAGGTCAGCATC	
*IgA*	F: ACCACGGCTCTGACTGTACC	S40610.1
	R: CGATGGTCTCCTTCACATCA	
*TLR2*	F: TGCCATTTCTCAAGGAGCTGT	NM_001161650
	R: GCTGATCGACATGGCCACTA	
*TLR4*	F: GATGCATCCCCAGTCCGTG	NM_001030693
	R: CCAGGGTGGTGTTTGGGATT	
*MyD88*	F: TGCAAGACCATGAAGAACGA	NM_001030962.3
	R: TCACGGCAGCAAGAGAGATT	
*TRAF6*	F: CACAGAGGAGACGCAGGGATA	XM_001235884.1
	R: AACAGATCGGGCACTCGTATTT	
*NF-κB*	F: TGGAGAAGGCTATGCAGCTT	NM_205134.1
	R: CATCCTGGACAGCAGTGAGA	
*IL-2*	F: AGTGCACCCAGCAAACTCTG	NM_204153.1
	R: TCCGGTGTGATTTAGACCCGT	
*IL-4*	F: GCTCTCAGTGCCGCTGATG	NM_0010079.1
	R: GAAACCTCTCCCTGGATGTCAT	
*IL-6*	F: GATCCGGCAGATGGTGATAA	NM_204628.1
	R: AGGATGAGGTGCATGGTGAT	
*IFN-γ*	F: AAAGCCGCACATCAAACACA	NM_205149.1
	R: GCCATCAGGAAGGTTGTTTTTC	
*TGF-β1*	F: GCCGACACGCAGTACACCAAG	NM_001318456.1
	R: GCAGGCACGGACCACCATATTG	
*CD3d*	F: TGTTGTCGCCACTGTCTTGCTG	NM_205512.1
	R: GTCCATCATTCCGCTCACCAAGG	
*CD4*	F: GATGGAGAGGTGTGGAGCAG	NM_204649
	R: CCTCCTTTCCTGCAATCCCA	
*TCR-β subunit*	F: ACTGGTATGCCTGGCCTCTGG	M81149.1
	R: CCACTCCTTCTGTCCTCTTCACAC	
*TCR-ζ subunit*	F: AGCTTAGCCAGGCCTCTGAA	AJ002317.1
	R: GGTGCCCAGCACATCGTATT	

a Primers designed using Primer Express software (Sangon Biotech, Shanghai, China).

b *GADPH*, glyceraldehyde-3-phosphate dehydrogenase; *MHCII*, major histocompatibility complex II; *iNOS*, inducible nitric oxide synthase; *AVBD2*, avian β defensins 2; *Lyz C*, lysozyme C; *pIgR*, polymeric immunoglobulin receptor; *IgA*, immune globulin A; *TLR2*, Toll-like receptor 2; *MyD88*, myeloid differentiation factor 88; *TRAF6*, TNF receptor associated factor 6; *NF-κB* = nuclear factor kappa-β; *TNF-α*, tumor necrosis factor α; *TGF-β1*, transforming growth factor-β1; *IL-2*, interleukin-2; *IFN-γ*, interferon-γ.

c F, forward; R, reverse.

### DNA extraction and high-throughput sequencing.

Bacterial DNA was extracted from ileal digesta with a QIAamp DNA Stool minikit (Qiagen, Inc., Valencia, CA) according to the manufacturer’s protocol. The concentrations of DNA extracts were measured on a NanoDrop 2000 spectrophotometer (Thermo Scientific, MA, USA). The V4 region of the bacterial 16S rRNA gene was amplified with the barcoded primer pair 515F/806R (515F: 5′-GTG CCA GCM GCC GCG GTA A-3′, 806R: 5′-GGA CTA CHV GGG TWT CTA AT-3′) according to previously described methods (91). After amplification, PCR products run on a 2% agarose gel and were purified using a QIAquick Gel Extraction Kit (Qiagen, Germany). Pyrosequencing of 16S rDNA was performed on an Illumina HiSeq2500 PE250 platform (Illumina, San Diego, USA) at Novogene Bioinformatics Technology Co. Ltd. (Beijing, China).

### Sequence processing and bioinformatics analysis.

Raw tags were generated by merging paired-end reads using FLASH software (v1.2.7) (92). High-quality clean tags were obtained by QIIME (v1.7.0) analysis (93), and chimera sequences were removed to obtain effective tags by using the UCHIME algorithm (94). Sequences were analyzed by UPARSE software (v7.0.1001) and clustered into operational taxonomic units (OTUs) at a similarity level of 97% (95). Each OTU was annotated with the Greengenes database (96). Rarefaction curve and Venn diagram were created using R software (v2.15.3). Analysis of microbial alpha diversity was conducted using QIIME software (93) with Python scripts. Beta diversity was evaluated by principal-component analysis to show the differences of bacterial community structures, and the significance of separation was tested via ANOSIM using R (v2.15.3). PICRUSt analysis was used to predict the functional potential of bacteria communities (97). OTUs were normalized by copy number, and metagenome prediction was further categorized into Kyoto Encyclopedia of Genes and Genomes at levels 2 and 3 (98).

### Statistical analysis.

SPSS 20.0 software was used to perform the statistical analysis on each group of data. The general linear model process was used for statistical analysis. When the interaction was significant, one-way analysis was used, and Duncan’s multiple comparison analysis was used for differences between treatments. *P < *0.05 was considered significant, and *P* values between 0.05 and 0.10 were classified as trends.

The Spearman rank correlation coefficient was used for the evaluation of the correlation analysis of the variables and microbiota in the broiler chickens.

### Data availability.

The data set generated and/or analyzed in the current research process can be obtained from the corresponding author upon reasonable request. The 16S rRNA gene amplicon sequencing results were submitted to the Sequence Read Archive of the NCBI (accession no. PRJNA769582).
